# Promoting Research under the Work Reform for Physicians

**DOI:** 10.31662/jmaj.2024-0066

**Published:** 2024-06-24

**Authors:** Soichiro Saeki, Reiko Okada, Wataru Horiguchi

**Affiliations:** 1 Center Hospital of the National Center for Global Health and Medicine, Tokyo, Japan; 2 Osaka Police Hospital, Osaka, Japan

**Keywords:** work-style reform, working conditions, postgraduate education, medical research, clinical research, Japan

Japan’s healthcare system is set to undergo considerable changes in 2024, with the implementation of work-style reform for physicians ^(1)^. Although the reform was initially planned for an earlier launch, it was postponed owing to concerns about insufficient physician numbers. The pressing need to reduce the excessive working hours of Japanese doctors has been emphasized by instances of “karoshi” (deaths induced by overwork) ^(2)^.

Notwithstanding its merits, this reform is not devoid of shortcomings. A salient concern is the ambiguity of working hours. As per the Ministry of Health, Labour, and Welfare, undertakings such as research and skill enhancement are not deemed labor unless explicitly mandated by a supervisor ^(1)^. Moreover, research hours were excluded from working hours ^(2)^. Although this provision facilitates time for self-improvement and research, it also implies that although academic meetings and research are required for medical specialty boards ^(3)^, such mandatory hours would not be compensated^(2)^. This could inadvertently stifle research within the Japanese medical fraternity, which necessitates the cultivation of research activities across a diverse spectrum of personnel and institutions, particularly considering Japan’s dwindling population ^(4)^.

To address the aforementioned challenges and continuously advance medical research, we suggest three strategies.

Primarily, providing physicians with schedule flexibility to accommodate other responsibilities is crucial. This requires the delegation of tasks to nonmedical professionals, which is a key aspect of the work-style transformation. Furthermore, medical institutions should establish procedures that allocate fixed research periods for physicians, similar to practices at some American hospitals.

Subsequently, obstacles to scholarly pursuits should be reduced. This involves implementing a system that fairly evaluates academic achievements, such as publications and conference presentations, and encouraging more healthcare institutions to develop strategies for teaching skills outside of physicians’ specialties, including research methodologies and statistical analysis.

Lastly, financial incentives are essential. A system that appropriately allocates compensation and overtime for research and mentorship hours, particularly during board certification, is necessary. One potential solution could be the development of an academic curriculum that follows the board certification program, focusing not only on clinical practice but also offering research incentives. Additionally, mentorship hours during research or manuscript preparation, often conducted by senior physicians ^(5)^, should be recognized as working hours and compensated accordingly.

The work-style reform will undoubtedly exert a considerable impact on hospital operations. However, clinical practice is not the sole domain affected. Flexibility in its administration is necessary to ensure that society can reap the benefits of its modifications.

## Article Information

### Conflicts of Interest

None

### Acknowledgement

The authors wish to thank their colleagues, especially those in clinical residency training, for their helpful discussions. The authors are also grateful to Paperpal (Cactus Communications Services Pte Ltd, Mumbai, India) and Microsoft Copilot (Microsoft Corporation, Redmond, WA, USA) for providing primary language editing. The views expressed in this manuscript are those of the authors and do not necessarily represent those of the authors’ institutions.

### Author Contributions

All authors took part in conceptualizing the manuscript. SS wrote the original draft, and the draft was critically edited by RO and WH. All authors read and approved the final draft of the manuscript. Artificial intelligence (AI) technology was used for the language editing process only, and the authors reviewed the contents after the language editing process. The author’s institution played no role in the conceptualization of this manuscript.

### Approval by Institutional Review Board (IRB)

Not applicable
